# Plasma hemoglobin and the risk of death in HIV/AIDS patients treated with antiretroviral therapy

**DOI:** 10.18632/aging.202987

**Published:** 2021-05-07

**Authors:** Dayong Wang, Xiangqing Hou, Xianghua Yu, Tao Wang, Zhenmiao Ye, Jushuang Li, Feifei Su, Chengnan Guo, Fang Peng, Shuzhen Zhao, Huihui Li, Jingjing Zuo, Dehua Su, Lina Zhao, Hemei Zhang, Xiangyang Chen, Ruoqiu Wang, Qipeng Xie, Chao Zheng, Guangyun Mao

**Affiliations:** 1 Wenzhou Center for Disease Control and Prevention, Wenzhou 325000, Zhejiang, China; 2 Department of Preventive Medicine, School of Public Health and Management, Wenzhou Medical University, Wenzhou 325035, Zhejiang, China; 3 Faculty of Health Sciences, University of Macau, Macau 999078, China; 4 Center on Evidence-Based Medicine and Clinical Epidemiology, School of Public Health and Management, Wenzhou Medical University, Wenzhou 325035, Zhejiang, China; 5 The Sixth People's Hospital of Wenzhou, Wenzhou 325035, Zhejiang, China; 6 School of Ophthalmology and Optometry, School of Biomedical Engineering, Wenzhou Medical University, Wenzhou 325000, Zhejiang, China; 7 The Second Affiliated Hospital of Wenzhou Medical University, Wenzhou 325000, Zhejiang, China; 8 The Second Affiliated Hospital of Zhejiang University School of Medicine, Hangzhou 310000, Zhejiang, China

**Keywords:** HB, HIV/AIDS associated death, interaction, C-index, net discrimination

## Abstract

Background: Previous studies concerning the effect of plasma hemoglobin (HB) and other factors that may modify the risk of death in people living with HIV/AIDS (PLHIV) treated with antiretroviral therapy (ART) are limited.

Results: Higher HB was independently linked to a lower death risk in PLHIV, with a decrease of 29% (13%, 43%) per standard deviation (SD) increment after adjusting for CD4, VL and other potential factors [hazard ratio (HR): 0.71, 95% confidence interval (CI): 0.57-0.87, P<0.001]. In addition, the addition of HB to the predictive model containing VL and CD4 significantly improved the C-index, by 0.69% (95% CI: 0.68%-0.71%), and net discrimination, by 0.5% (95% CI: 0.0%-1.6%, P=0.040), when predicting the death risk of PLHIV.

Conclusions: A lower level of HB was an independent risk factor for HIV/AIDS-associated death in PLHIV. HB combined with VL and CD4 may be an appropriate predictive model of the death risk of PLHIV.

Materials and methods: A propensity-score matching (PSM) approach was applied to select a total of 750 PLHIV (150 deceased and 600 living) from the AIDS prevention and control information system in the Wenzhou area from 2006 to 2018. Multivariable Cox proportional hazards regression models were formulated to estimate the effect of HB. The predictive performance improvement contributed by HB was evaluated using the C-index and net reclassification improvement.

## INTRODUCTION

Human immunodeficiency virus (HIV) infection and acquired immune deficiency syndrome (AIDS) are threats to human health and have presented a great global public health burden in the past several decades. It has been suggested that approximately 32 million people have suffered from HIV/AIDS worldwide to date [1]. To efficiently limit the global AIDS epidemic, a program comprising three 90% prevention and control measures (90% diagnosis, 90% treatment and 90% effectiveness by 2020, 90-90-90) was suggested by the United Nations Programme on AIDS (UNAIDS). At the end of 2015, China achieved proportions of 68%, 67% and 91%, respectively, for these three targets [2]. It is estimated that more than 19.5 million people living with HIV (PLHIV) have received standard antiretroviral therapy (ART) as of 2016. While PLHIV with successful virus suppression have a life expectancy similar to that in the general population [3], those with higher death risk usually have worse quality of life. Therefore, timely identification of potential biomarkers that can indicate higher AIDS-related mortality would be valuable for deploying medical resources appropriately and establishing early healthcare interventions [4].

Metabolic biomarkers such as albumin (ALB) and alkaline phosphatase (AP) are closely related to both mortality of PLHIV and progression of HIV infection [5–8]. Notably, anemia is associated with the mortality of PLHIV, possibly due to hematological abnormalities [9–12]. Several previous studies have been conducted to determine the associations between death risk and HB [13, 14], which is not only helpful for better understanding the effect of typical laboratory biomarkers on the death risk of PLHIV but also important for clinical records of HIV/AIDS progression. Nevertheless, research that concentrates on accurately quantifying the effect of HB on the death risk of PLHIV is limited. In addition, few studies have been conducted to examine potential modifiers of the association between HB and the mortality of PLHIV.

In this nested case-control study, we comprehensively investigated the effects of HB on the death risk of PLHIV treated with ART. In addition, we further assessed the discrimination and reclassification ability of a predictive model by adding HB to traditional risk factors, including CD4 and VL.

## MATERIALS AND METHODS

### Study design

The present study was based on our previous work [15]. Briefly, in this nested case-control study, we observed that 150 eligible people living with HIV (PLHIV) receiving antiretroviral therapy (ART) died in a retrospective cohort of the AIDS Prevention and Control Information Subsystem (AIDS-PCIS). This system was developed in 2006 by the Chinese Centre for Disease Control and Prevention (CCDC) and aimed at carrying out the surveillance and management of HIV infections [16]. To improve the robustness and reliability of our conclusions, a propensity-score matching (PSM) approach was applied to perform a case-control match at a ratio of 1:4 to identify the participants, in which one case (deceased PLHIV) was matched by age and gender with 4 controls (living PLHIV).

### Data collection

Data on demographic characteristics, clinical features, laboratory assessments and factors were uploaded to AIDS-PCIS in real-time. Participants enrolled in the cohort were regularly followed up every 3 months. Updated laboratory signs, including CD4^+^ T lymphocytopenia (CD4), CD8^+^ T lymphocytopenia (CD8), viral load (VL), white blood cells (WBCs), platelets (PLTs), hemoglobin (HB), serum creatinine (CR), triglycerides (TGs), serum total cholesterol (TC), fasting plasma glucose (FPG), alanine aminotransferase (ALT), aspartate transaminase (AST), total bilirubin (TBIL) and others, were collected at each follow-up.

According to the inclusion and exclusion criteria described in our previous study [15], a total of 3733 PLHIV treated with ART in the Wenzhou area from 2006 to 2018 were extracted from AIDS-PCIS. Among them, 150 died and were defined as cases, and 600 living PLHIV were matched as controls. Therefore, 750 PLHIV (blocks of 150; 150 cases and 600 controls) were included in the final analysis.

### Statistics analysis

A logistic regression model was applied to perform propensity-score matching (PSM) at a ratio of 1:4 to match the cases (deceased PLHIV) and controls (living PLHIV) by age and sex. Continuous data are presented as the mean ± standard deviation (SD) or median (1^st^ quartile, 3^rd^ quartile) depending on their distributions. Categorical variables are described as frequencies (percentages). To robustly characterize the effect of HB on the risk of death in PLHIV treated with ART, a restrictive cubic spline regression model was performed first to examine the possible exposure-response relationship between HB level and the death risk of PLHIV. Subanalysis stratified by age, sex, body mass index (BMI, <25.0 *vs.* ≥25.0), CD4 (<200 *vs*. ≥200), CD8 [<857.4 (median) *vs*. ≥857.4], WBC [<5.29 (median) *vs*. ≥5.29], TC [<4.08 (median) *vs*. ≥4.08], ALT [<24.6 (median) *vs*. ≥24.6], TBIL [<10.6 (median) *vs*. ≥10.6] and FPG [<7.0 (median) *vs*. ≥7.0] was further performed. The individual effect of HB on PLHIV mortality was comprehensively quantified by hazard ratio (HR) and 95% confidence interval (CI) with HB as a continuous variable [scaled to per-standard deviation (SD) increase] or as a categorical variable (quartiles) based on multivariable Cox proportion hazard regression models. To properly screen potential confounding factors, variables with p-values less than 0.2 [17] in the association analysis on the presence of death were considered covariates.

Furthermore, to determine the impacts of potential modifiers on the effects of HB, we performed a broad exploration of the interaction between HB and some baseline characteristics, HIV progression indicators, and serological biomarkers. Both CD4 and VL were classified into two categories with 200 cells/μL, a widely accepted criterion of CD4 category, and VL classification standards as the cutoff points. To assess the effect of age category on the associations between markers of HIV progression and metabolic biomarkers, the patients were categorized into two groups using 50 as the cutoff value as in previous similar research [13].

To quantify the additive improvement of the predictive ability of HB, we further developed a new model based on conventional risk factors such as CD4 and VL. We also carefully assessed the additional improvement of the model performance via the C-index and continuous net reclassification improvement (NRI) or integrated discrimination improvement (IDI). We calculated continuous NRI rather than NRI because no widely accepted death risk cutoff value could be selected.

All tests were two-sided, and *P*<=0.05 was considered to be significant. All data management and statistical analyses were performed using Stata/MP 15.1 (Copyright 1985-2017 State Corp Institute Inc.). Figures were drawn with R-studio 1.1.456 for Windows (Copyright 2009-2018 R-Studio, Inc.).

### Ethical statement

The data utilized in the present study were extracted from AIDS-PCIS, which was established by the National Centre for AIDS/Sexually Transmitted Disease Control and Prevention of the CCDC to generate the HIV/AIDS epidemic database with the continued enrollment of HIV-infected persons [18, 19]. The protocol of the current study was also approved by the ethical review board of the Wenzhou Centre for Disease Control and Prevention.

## RESULTS

A total of 750 participants (150 deceased and 600 living) were included in the analysis, and the majority were male (84.4%) and of Han nationality (97.9%). The average age was 50.3±16.8 years, and 52% were under 50 years old. The median (1^st^ quartile-3^rd^ quartile) baseline CD4 and CD8 counts were 207.0 (88.0-317.0) and 857.4 (546.0-1267.0) cells/μL, respectively. Separate proportions of baseline VL were 78.40% for less than 200 copies/mL, 2.67% for 200~1000 copies/mL and 18.93% for over 1000 copies/mL. In addition, the median (1^st^ quartile-3^rd^ quartile) baseline HB was 136.0 (118.8-150.0) g/L.

### Baseline characteristics between deceased and living PLHIV

As shown in Table 1, deceased PLHIV were more likely to have significantly higher VL, FPG and AST levels than living PLHIV, which indicated that diabetes and hepatic insufficiency might be harmful to PLHIV. In contrast, BMI, CD4, CD8, WBC, HB and TBIL of the cases were significantly lower than those of the controls. When compared with their counterparts, the deceased PLHIV were more likely farmers, at the stage of advanced WHO, progressed to AIDS status, hospital visits for HIV/AIDS and much more likely to be combined with tuberculosis (TB) infection. We also observed that infection through the men who have sex with men (MSM) pathway was lower and that nonmarital heterosexual transmission was more common in the deceased participants than in the living participants. Other characteristics, such as hepatitis B virus (HBV) infection, marital status, sexually transmitted disease (STD) history, etc. between the cases and controls were similar or not significantly different. Moreover, gender and age were very comparable between the deceased and living patient groups, which suggested that the PSM approach worked well and that the impacts due to known and unknown confounding biases on the effect of HB would be avoided to some extent.

**Table 1:** Characters of PLHIV death and live subjects.

**Variables**	**Total** **(n=750)**	**Alive** **(n=600)**	**Dead** **(n=150)**	***p-value***
**Discrete variables**				
Gender				0.880
Man	633(84.4)	507(84.5)	126(84.0)	
Woman	117(15.6)	93(15.5)	24(16.0)	
Viral Load, copies/mL				<0.001
<200	588(78.4)	557(92.8)	31(20.7)	
200~1000	20(2.7)	17(2.8)	3(2.0)	
>=1000	142(18.9)	26(4.3)	116(77.3)	
TB				0.003
Yes	33(4.4)	19(3.2)	14(9.3)	
No	698(93.1)	567(94.5)	131(87.3)	
Unknown	19(2.5)	14(2.3)	5(3.3)	
WHO Stage				<0.001
I	390(52.0)	349(58.2)	41(27.3)	
II	70(9.3)	57(9.5)	13(8.7)	
III	222(29.6)	159(26.5)	63(42.0)	
IV	68(9.1)	35(5.8)	33(22.0)	
HBV				0.190
Yes	96(12.8)	72(12.0)	24(16.0)	
No	654(87.2)	528(88.0)	126(84.0)	
Infection pathway				0.006
NMHR	438(58.4)	335(55.8)	103(68.7)	
MSM	223(29.7)	194(32.3)	29(19.3)	
Others	89(11.9)	71(11.8)	18(12.0)	
Marital status				0.584
Married	390(52.0)	315(52.5)	75(50.0)	
Unmarried	360(48.0)	285(47.5)	75(50.0)	
Occupation				0.001
Farmer	190(25.3)	142(23.7)	48(32.0)	
Business	144(19.2)	130(21.7)	14(9.3)	
House keeping	164(21.9)	135(22.5)	29(19.3)	
Workers	105(14.0)	86(14.3)	19(12.7)	
Others	147(19.6)	107(17.8)	40(26.7)	
History STD				0.526
Yes	118(15.7)	97(16.2)	21(14.0)	
No	447(59.6)	360(60.0)	87(58.0)	
Unknown	185(24.7)	143(23.8)	42(28.0)	
Participants category				0.403
Fixed population	584(77.9)	471(78.5)	113(75.3)	
Floating population	166(22.1)	129(21.5)	37(24.7)	
Race				0.658
Han	734(97.9)	586(97.7)	148(98.7)	
Others	16(2.1)	14(2.3)	2(1.3)	
Education				0.204
Illiterate or elementary school	278(37.1)	216(36.0)	62(41.3)	
Senior middle school	79(10.5)	70(11.7)	9(6.0)	
Junior high school	116(15.5)	93(15.5)	23(15.3)	
College and above	277(36.9)	221(36.8)	56(37.3)	
Disease stage				<0.001
AIDS	405(54.0)	266(44.3)	139(92.7)	
HIV	345(46.0)	334(55.7)	11(7.3)	
Origin of identification				<0.001
Others	108(14.4)	90(15.0)	18(12.0)	
CDC	185(24.7)	168(28.0)	17(11.3)	
Hospital	457(60.9)	342(57.0)	115(76.7)	
**Continuous variables**				
Age, year	49.5(37.5,63.2)	49.7(37.5,63.3)	48.3(38.2,63.0)	0.978
Body mass index, kg/m^2^	21.3(19.3,23.5)	21.5(19.6,23.9)	20.6(18.3,21.9)	<0.001
CD4+ T-lymphocyte count, cells/μL	207.0(88.0,317.0)	244.8(136.0,347.0)	60.0(24.0,115.3)	<0.001
CD8+ T-lymphocyte count, cells/μL	857.4(546.0,1267.0)	917.5(636.5,1330.2)	570.9(323.0,960.0)	<0.001
White blood cell, 10^9^/L	5.3(4.2,6.7)	5.3(4.3,6.7)	4.8(3.6,7.1)	0.054
Platelet, 10^9^/L	187.0(144.0,227.4)	185.2(145.0,224.0)	202.0(141.0,242.0)	0.241
Hemoglobin, g/L	136.0(118.8,150.0)	140.0(123.0,152.0)	116.5(98.0,130.1)	<0.001
Creatinine, μmol/L	70.0(58.3,82.0)	70.0(59.0,81.6)	71.0(58.0,86.0)	0.707
Triglyceride, mmol/L	1.5(1.0,2.4)	1.4(1.0,2.3)	1.6(1.0,2.5)	0.310
Total cholesterol, mmol/L	4.1±0.9	4.2±0.9	3.7±1.1	<0.001
Fasting plasma glucose, mmol/L	5.3(4.6,6.6)	5.3(4.6,6.5)	5.6(4.7,8.6)	0.043
Aspartate transaminase, U/L	24.6(19.0,34.1)	23.7(19.0,32.0)	30.0(22.0,43.0)	<0.001
Alanine aminotransferase, U/L	21.0(15.0,34.2)	21.0(15.0,32.0)	24.0(15.0,38.0)	0.173
Total bilirubin, μmol/L	10.6(7.5,15.4)	11.1(7.9,15.8)	8.6(6.8,14.5)	0.001

Note: Age was described as median (1st quartile, 3rd quartile) as its distribution was skewed and Mann-Whitney U test was applied to compare the difference between two groups; Categorical data were presented with number (%) and chi-square tests or Fisher’s exact test were used to compare the differences between alive and dead participants.

NMHR: Non-marital heterosexual transmission; MSM: men who have sex with men; CDC: center for disease prevention and control.

### Association of hemoglobin with death risk

**Figure 1:**
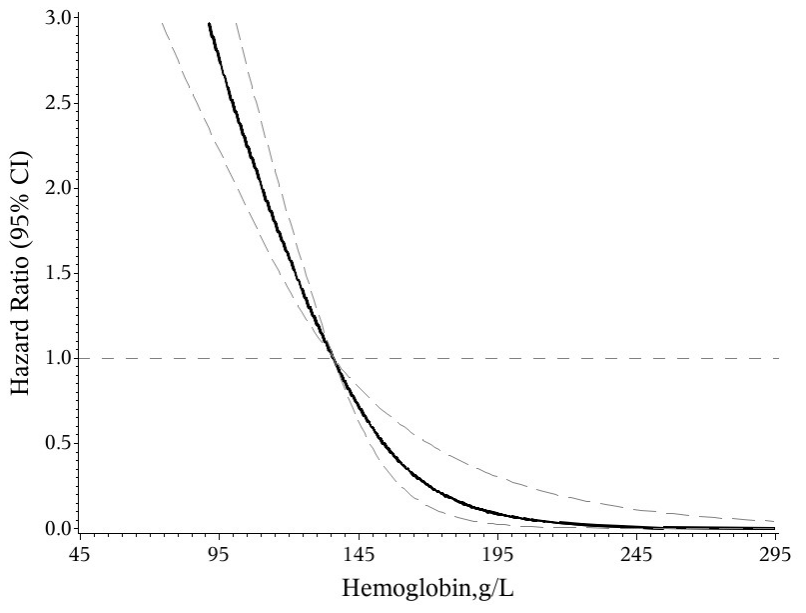
Relationship of hemoglobin level with the death risk of PLHIV received regular ART treatment.

**Figure 2:**
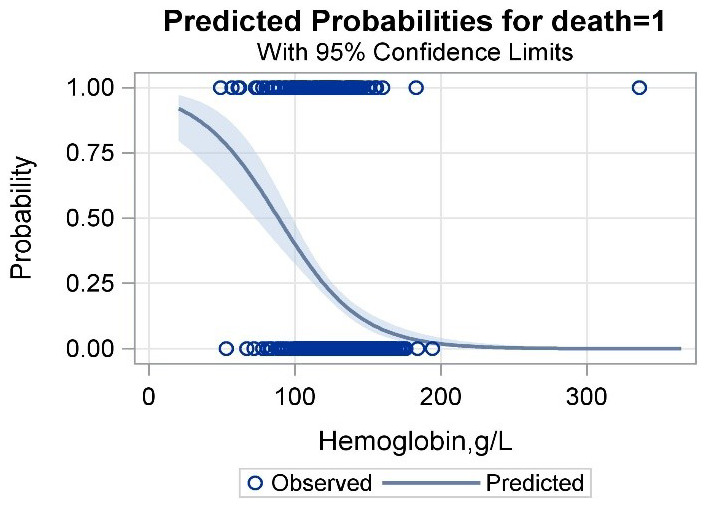
Predictive effect of hemoglobin on the death risk of PLHIV received regular ART treatment.

Based on the restrictive cubic spline regression model, we detected a monotonically decreased exposure-response curve between baseline HB and HR (Figure 1), which clearly revealed that elevated HB levels were related to a decreased risk of death in PLHIV treated with ART, especially in those with HB less than 200 g/L. The predictive effect diagrams of HB on the mortality of PLHIV, depending on a multivariable logistic regression model, also suggested that HB was negatively linked to the risk of death and might be a potentially appropriate predictor with good performance (Figure 2). Table 2 presents the individual effect of HB on the death risk of PLHIV in two ways. When HB was considered a continuous variable, the adjusted death risk decreased by an average of 29% (HR: 0.71, 95% CI: 0.57-0.87, P<0.001) with a per SD increase in HB intensity. Furthermore, when HB was considered a categorical variable (quartile), the sequential proportions of deaths in the 1^st^ (HB<118.8 g/L), 2^nd^ (HB 118.8-136.0 g/L), 3^rd^ (HB 136.1-149.7 g/L) and 4^th^ (HB>=150.0 g/L) quartiles of HB were 43.32%, 20.63%, 10.81% and 5.29%, respectively. Compared to that in the PLHIV with the lowest quartile of HB, the death risk of PLHIV in the other 3 quartiles was decreased by 27% (HR: 0.73, 95% CI: 0.46-1.16, P=0.182), 45% (HR: 0.55, 95% CI: 0.31-0.96, P=0.036) and 64% (HR: 0.36, 95% CI: 0.16-0.80, P=0.012), respectively. An obvious linear trend was found between the decreased risk of death and elevated HB level in PLHIV treated with ART (P=0.004).

**Table 2:** Risk of death of PLHIV associated with hemoglobin.

**HB, g/L**	**n**	**Death,** **# (%)**	***Crude***			***Adjusted^¶^***	
			***HR (95% CI)***	***P-value***		***HR (95% CI)***	***P-value***
Per SD increment (24.8)	750		0.53(0.47,0.60)	<0.001		0.71(0.57,0.87)	<0.001
Quartile							
Q_1_(<118.0)	187	81(43.32)	1.00(1.00,1.00)	Ref.		1.00(1.00,1.00)	Ref.
Q_2_(118.8-136.0)	189	39(20.63)	0.42(0.28,0.61)	<0.001		0.73(0.46,1.16)	0.182
Q_3_(136.1-149.7)	185	20(10.81)	0.22(0.14,0.36)	<0.001		0.55(0.31,0.96)	0.036
Q_4_(≥150.0)	189	10(5.29)	0.11(0.06,0.20)	<0.001		0.36(0.16,0.80)	0.012
P for trend				<0.001			0.004
Anemia***^β^***							
No	567	69(12.17)	1.00(1.00,1.00)	Ref.		1.00(1.00,1.00)	Ref.
Yes	183	81(44.26)	4.21(3.05,5.80)	<0.001		1.78(1.17,2.71)	0.007

¶: Adjusted for VL, TB, WHO stage, HBV, infection pathway, occupation, disease stage, origin of identification, BMI, CD4, CD8, WBC, TC, FPG, ALT, AST and TBIL.

β: Anemia is defined as HB levels less than 120 g/L in man or HB levels less than 110 g/L in woman.

In addition, to further explore the effect of HB on mortality, we split the 750 PLHIV into anemia and non-anemia groups according to their HB levels. The results showed that anemic PLHIV had a significantly higher death risk (HR: 1.78, 95% CI: 1.17-2.71, P=0.007). We also performed an additional sensitivity analysis with different matching ratios (1:1, 1:2, 1:3 and 1:4) of PSM (Supplementary Table 1). These results clearly confirmed that a lower HB level was an independent risk factor for death in PLHIV receiving ART.

### Subgroup analysis for detecting potential modifiers

Table 3 presents the subgroup analysis results regarding the effect of HB (per SD increment) on the death risk of PLHIV stratified by age, sex, VL, TC and others. The effect of HB was clearly modified by age (P_interaction_=0.043), TC (P_interaction_=0.021) and VL (P_interaction_=0.062). PLHIV over 50 years old with TC greater than 4.08 mmol/L or VL less than 200 copies/mL would be more likely to have a larger reduction in death risk than their counterparts with the same elevation of HB. However, the impacts of other variables, including sex, BMI, CD4, CD8, WBC, ALT, TBIL and FPG, on the effects of HB were not obvious.

**Table 3:** The association between hemoglobin (per SD increment) and risk of death of PLHIV in various subgroups.

**Variables**	**n**	**Death, # (%)**	***HR(95% CI) ^¶^***		***P for interaction^¶^***
Demographic Characteristic					
Sex					0.495
Man	633	126(19.91)	0.76(0.61,0.95)		
Woman	117	24(20.51)	0.51(0.27,0.97)		
Age, year					0.043
<50	390	79(20.26)	0.78(0.59,1.05)		
≥50	360	71(19.72)	0.66(0.46,0.95)		
BMI, kg/m2					0.112
<25.0	639	135(21.13)	0.74(0.60,0.92)		
≥25.0	111	15(13.51)	0.50(0.08,3.10)		
HIV progress					
CD4, cells/μL					0.774
<200	365	132(36.16)	0.68(0.55,0.84)		
≥200	385	18(4.68)	0.83(0.39,1.75)		
CD8, cells/μL					0.900
<857.4	375	107(28.53)	0.71(0.57,0.90)		
≥857.4	375	43(11.47)	0.73(0.43,1.27)		
Viral load, copies/mL					0.062
<200	588	31(5.27)	0.57(0.37,0.86)		
≥200	162	119(73.46)	0.69(0.54,0.88)		
Serological biomarker					
White blood cell, 109/L					0.447
<5.29	375	87(23.20)	0.60(0.45,0.78)		
≥5.29	375	63(16.80)	0.85(0.60,1.22)		
Total cholesterol, mmol/L					0.021
<4.08	375	97(25.87)	0.78(0.60,1.00)		
≥4.08	375	53(14.13)	0.61(0.42,0.88)		
Alanine aminotransferase, U/L				0.747
<24.6	375	49(13.07)	0.76(0.49,1.17)		
≥24.6	375	101(26.93)	0.70(0.55,0.89)		
Total bilirubin, μmol/L					0.179
<10.6	375	94(25.07)	0.73(0.55,0.96)		
≥10.6	375	56(14.93)	0.62(0.42,0.91)		
Fasting plasma glucose, mmol/L				0.140
<7.0	583	102(17.50)	0.76(0.58,1.00)		
≥7.0	167	48(28.74)	0.58(0.39,0.87)		

¶: Adjusted for VL, TB, WHO stage, HBV, infection pathway, occupation, disease stage, origin of identification, BMI, CD4, CD8, WBC, TC, FPG, ALT, AST and TBIL.

### Incremental death risk prediction

We observed that the C-index increased by 0.69% (95% CI: 0.68%-0.71%) when HB was added to the predictive model including CD4 and VL (Supplementary Table 2). Furthermore, we also detected marginally significant improvements in continuous NRI by 0.110 (95% CI: -0.008- 0.185, P=0.08) and absolute improvement of IDI in the mean discrimination slope by 0.005 (95% CI: 0.000-0.016, P=0.04) (Supplementary Table 3).

## DISCUSSION

The present study carefully examined the effect of baseline HB on the death risk of PLHIV and some potential modifiers of this effect. This study also investigated the additional improvement in the prediction of the death risk of PLHIV by adding HB to the model. Overall, we report a negative association between HB levels and the risk of death in PLHIV. Such an association was not altered by baseline demographic factors except for age and serum total cholesterol (TC), after adjusting for other confounders. In addition, we determined that HB will be helpful for improving the performance of a prognostic model including CD4 and VL in the prediction of PLHIV death risk. Our findings will contribute to better understanding the associations between baseline HB levels and mortality of PLHIV.

Our results clearly demonstrate that a higher level of HB is independently associated with a reduction in HIV/AIDS-related death risk. To the best of our knowledge, this is the first report focusing on quantitatively estimating the associations between baseline HB and death risk of PLHIV in a large-scale population-based cohort from China. Consistent with our findings, a previous study also suggests that an increased level of HB has a close relationship with the decreased death risk of PLHIV [5]. We postulate that some clinically relevant evidence can explain such an association. First, chronic inflammation contributes to the development of anemia and is observed in a general population as well as in PLHIV. A previous report [9] demonstrated that hematological abnormalities obviously increase the mortality of PLHIV with anemia. Second, PLHIV with long-term anemia often also experience nutritional deficiencies or irregular lifestyles and an increased likelihood of complications due to insufficient treatment. Therefore, PLHIV with HB dysfunction usually have a higher risk of mortality to some extent than their counterparts. In addition, while ART is effective in repressing the development of anemia, many HIV-infected individuals still have unresolved anemia or develop anemia.

Available evidence reveals that the risk of death in PLHIV is usually affected by a number of demographic characteristics and laboratory factors, including CD4, ALB, TC, age, sex and others [20]. Therefore, whether they are potential modifiers of the association between HB and mortality of PLHIV should be an essential addition to this research. Interestingly, we detected that age may be a strong modifier of the effect of HB since the association between HB and the risk of death is much more robust in PLHIV aged over 50 years. It has been reported that the prevalence of anemia increases rapidly in people older than 50 years [21]. We speculate that hemoglobin abnormalities are very common in seniors. When compared with the general population, HIV-infected individuals are more likely to have long-term anemia, which significantly increases the mortality in PLHIV [22]. Furthermore, we also detected that serum TC may be another modifier of the effect of HB on the death risk of PLHIV. The effect of HB is much more evident in PLHIV whose TC is more than 4.08 mmol/L. Bijker R and colleagues demonstrated that cardiovascular disease (CVD) has become an increasingly significant contributor to morbidity and mortality among PLHIV, who are prone to developing coronary heart disease during long-term ART initiation [23]. The identified plausible biological mechanisms, including endothelial dysfunction and arterial inflammation, can provide reasonable explanations for the association [24]. It is well known that participants with CVD often have elevated TC, which shares a similar biological mechanism with HB with regard to the mortality of PLHIV because anemia has been demonstrated to be a predominant contributor to chronic inflammation [25]. Of note, we were not able to find a similar interaction between other laboratory factors and HB, which may be due to their different cellular characteristics and mechanisms in the prognosis of PLHIV.

Our previous work suggested that HB might be a good predictor of death risk in PLHIV treated with ART. However, its contribution to the improvement of current risk predictions was unclear. In this study, we observed that the C-index (95% CI) was 0.912 (0.894, 0.931) for a model including VL, CD4 and HB and 0.906 (0.885, 0.926) for a model including only CD4 and VL. The model including HB performed statistically better than another model containing VL and CD4, with an incremental C-index (95% CI) of 0.69% (0.68%, 0.71%) (P=0.002). In addition, the improvement of IDI in the prediction of PLHIV mortality provided by HB also supports the significant benefit of considering HB values. Moreover, it is well known that HB is a routine laboratory assessment and can be readily applied in almost all hospitals. In addition, the inclusion of too few predictors in a model can lead to underfitting. Our findings suggest that HB plays an important and independent role in the prediction of the risk of death in PLHIV who received ART treatment and greatly improves the performance of predictive models including HB, CD4 and VL. Therefore, we highly recommend that HB should be an important predictor in the prognosis of PLHIV death risk.

Some strengths of the present study are highlighted as follows: (1) To improve the robustness and reliability of our conclusion, participants were determined via a propensity-score matching (PSM) approach at a ratio of 1:4. (2) Associations between the occurrence of death and potential prognostic factors including both clinical and laboratory data were comprehensively analyzed. (3) The effects of HB on the performance, discrimination and reclassification of the predictive model were thoroughly assessed based on a separate validation cohort set that was randomly selected from 750 enrolled PLHIV and independent of the training cohort set. (4) We also thoroughly assessed the association of HB with the mortality of PLHIV and the influence of some potential modifiers on the association via subgroup analysis. Additionally, several limitations need to be considered. First, all predictors used in the current study are derived from the baseline data, but some of them may be time-dependent, which may partly affect the strength of the association. Second, the study site is limited to the Wenzhou area of China, which might induce potential selection bias to some extent. Additional longitudinal studies in other places are needed to verify our findings.

## CONCLUSIONS

We demonstrate that a higher HB level is significantly related to a decreased risk of death in PLHIV receiving ART treatment. The protective effect of HB on the death risk of PLHIV will be obviously modified by age and serum TC as well as VL. Our findings also suggest that HB plays an important role in predicting the risk of death in PLHIV and improves the performance of a predictive model including CD4 and VL. We believe that our findings are beneficial for deploying medical resources properly and implementing early health-care administration of PLHIV.

## Supplementary Material

Supplementary Tables
