# Supplementary material for: Plasma hemoglobin and the risk of death in HIV/AIDS patients treated with antiretroviral therapy
Source: Aging (Albany NY). 2021 May 7;13(9):13061–72. doi: 10.18632/aging.202987 (PMC8148493; doi:10.18632/aging.202987)
Supplement: Supplementary Tables [file aging-13-202987-s001.pdf]

SUPPLEMENTARY TABLES

Supplementary Table 1. Sensitivity analysis for different matching ratio.

HB, g/L	n	Death, # (%)	*Crude*		*Adjusted*¶	
			HR (95% CI)	*P-value*	*HR (95% CI)*	*P-value*
Anemia$^{\beta}$ (1:4)						
No	567	69(12.17)	1.00(1.00,1.00)	Ref.	1.00(1.00,1.00)	Ref.
Yes	183	81(44.26)	4.21(3.05,5.80)	<0.001	1.78(1.17,2.71)	0.007
Anemia (1:3)						
No	428	69(16.12)	1.00(1.00,1.00)	Ref.	1.00(1.00,1.00)	Ref.
Yes	172	81(47.09)	3.40(2.46,4.69)	<0.001	1.65(1.08,2.50)	0.020
Anemia (1:2)						
No	309	69(22.33)	1.00(1.00,1.00)	Ref.	1.00(1.00,1.00)	Ref.
Yes	141	81(57.45)	3.20(2.32,4.42)	<0.001	1.68(1.10,2.55)	0.016
Anemia (1:1)						
No	186	69(37.10)	1.00(1.00,1.00)	Ref.	1.00(1.00,1.00)	Ref.
Yes	114	81(71.05)	2.65(1.92,3.66)	<0.001	1.52(1.00,2.33)	0.055

¶: Adjusted for VL, TB, WHO stage, HBV, infection pathway, occupation, disease stage, origin of identification, BMI, CD4, CD8, WBC, TC, FPG, ALT, AST and TBIL.
β: Anemia is defined as HB levels less than 120 g/L in man or HB levels less than 110 g/L in woman.

Supplementary Table 2. Change of C-index and after adding information of HB to model including CD4 and VL.

Variable	C-index (95% CI)	C-index increment (95% CI)
CD4+VL	0.906(0.885, 0.926)	— —
CD4+VL+HB	0.912(0.894, 0.931)	0.0069(0.0068, 0.0071)$^{\alpha}$

α:P<0.05.

Supplementary Table 3. Change in PLHIV's death risk of reclassification after adding information of HB to model including CD4 and VL.

Variable	CNRI (95%CI)	IDI (95%CI)
CD4+VL	— —	— —
CD4+VL+HB	0.110(-0.008, 0.185)	0.005(0.000, 0.016)$^{\alpha}$

α:P<0.05.
